# Maternal Loss of miRNAs Leads to Increased Variance in Primordial Germ Cell Numbers in *Drosophila melanogaster*

**DOI:** 10.1534/g3.113.007591

**Published:** 2013-09-01

**Authors:** Jan-Michael Kugler, Ya-Wen Chen, Ruifen Weng, Stephen M. Cohen

**Affiliations:** * Institute of Molecular and Cell Biology, Singapore 138673, Republic of Singapore; † Department of Biological Sciences, National University of Singapore, Singapore 119613, Republic of Singapore

**Keywords:** microRNA, phenotypic trait variance, primordial germ cell development

## Abstract

MicroRNAs (miRNAs) are posttranscriptional regulators of gene expression that may act as buffering agents to stabilize gene-regulatory networks. Here, we identify two miRNAs that are maternally required for normal embryonic primordial germ cell development in *Drosophila melanogaster*. Embryos derived from *miR-969* and *miR-9c* mutant mothers had, on average, reduced germ cell numbers. Intriguingly, this reduction correlated with an increase in the variance of this quantitative phenotypic trait. Analysis of an independent set of maternal mutant genotypes suggests that reduction of germ cell number need not lead to increased variance. Our observations are consistent with the hypothesis that *miR-969* and *miR-9c* contribute to stabilizing the processes that control germ number, supporting phenotypic robustness.

In most metazoan species, genetic information is propagated through generations by germ cells. Typically, primordial germs cells (PGCs) are specified in early embryonic development, either through inductive signals or through maternal inheritance of cell fate determinants (Extavour and Akam 2003). Following their formation, PGCs migrate through the embryo to settle in the somatic gonad (Richardson and Lehmann 2010). In *Drosophila melanogaster*, PGCs are specified through maternal inheritance of a specialized cytoplasm called germ plasm (Mahowald 2001). The germ plasm has been shown to be necessary and sufficient for PGC formation in *Drosophila*. Some components of the germ plasm also are essential for normal development of somatic posterior structures. The germ plasm is assembled in the posterior pole of the oocyte and persists into the early embryo. During the syncytial nuclear divisions in the early embryo, a few nuclei migrate to the posterior pole of the embryo and associate with the germ plasm. These nuclei and the germ plasm are incorporated in the PGCs as they cellularize. PGCs then divide several times before they start migrating toward the somatic gonadal structures.

Many genes have been identified that are maternally required for normal PGC development. The establishment of the future body axes in *Drosophila* requires polarized distribution of mRNAs and proteins within the oocyte (Lasko 2011). Tight translational regulation of localized mRNAs is essential. Thus, misregulation of the pathways that polarize the oocyte and distribute cell fate determinants can impact embryonic PGC formation (*e.g.*, Liu *et al.* 2003; Thomson *et al.* 2008; Kugler *et al.* 2010). However, PGC development also can be affected by maternally acting genes that regulate nuclear divisions and nuclear migration in the syncytial embryo (Yohn *et al.* 2003) or that affect germ cell migration (Kunwar *et al.* 2003).

We investigated whether microRNAs (miRNAs) might be maternally involved in regulating embryonic PGC development. microRNAs are posttranscriptional regulators of gene expression that are thought to serve as buffering agents to minimize noise and to stabilize gene regulatory networks (Herranz and Cohen 2010; Ebert and Sharp 2012). Given their role as dampeners of gene expression, it has been suggested that miRNAs might help to manage biological noise and confer robustness to developmental programs. Attractive as the idea is, there has been little quantitative experimental evidence supporting a role for microRNAs in managing biological variation. *Drosophila miR-7* has been shown to be essential to stabilize multiple regulatory networks under conditions of environmental flux (Li *et al.* 2009). A recent study reported a correlation between differences in the expression pattern of *Drosophila miR-92a* with the intraspecific variation of a quantitative phenotypic trait (Arif *et al.* 2013). However, this study did not address whether the miRNA acts to stabilize the trait.

Here, we identify two Drosophila miRNAs, *miR-9c* and *miR-969*, whose maternal loss causes an increase in the variance of germ cell numbers, consistent with the idea that these miRNAs stabilize maternal pathways governing embryonic germ cell development.

## Materials and Methods

The miRNA mutants were generated as shown in Supporting Information, Figure S1 (Chen *et al.* 2011). The *miR-969**^KI^* mutant allele is a Gal4 “knock-in” allele (Teleman *et al.* 2006). *Df(1)BSC352* is a genomic deficiency uncovering the *miR-969* locus. *T-969@Fb* is an UAS transgene allowing expression of the *miR-969* hairpin (Szuplewski *et al.* 2012). The *miR-9c**^KO^* mutant allele is a deletion mutation for which the mini-white cassette initially replacing the miRNA was excised by lox-CRE–mediated *cis*-recombination. *T-9c@Fb* is an UAS transgene allowing expression of the *miR-9c* hairpin (Szuplewski *et al.* 2012). All mutants were confirmed by genomic polymerase chain reaction and/or sequencing. The initial screen for germ line phenotypes was performed by crossing miRNA mutants to genomic deficiencies (Roote and Russell 2012) uncovering the miRNA loci.

Embryos were collected, fixed, and stained as described (Kugler *et al.* 2010). The PGCs were counted at embryonic stage S10 with an Axioimager microscope (Zeiss) at 200× magnification. Data from the primary screen (not shown) were validated in separate experiments including all proper controls and rescue conditions. Six batches of 10 embryos each were counted separately per genotype. Raw data are presented in Table S1. Averages and SDs were calculated over the whole population of 60 embryos. Variance was calculated for each batch of 10 embryos, and their average and SD were plotted using Excel (Microsoft). Importantly, the variance calculated over the entire population of 60 embryos was essentially the same as the average variance of the subpopulations of 10 embryos. The original data of Table 2 in the article by Kugler *et al.* 2010 were analyzed by calculating the variance of each data point and plotting it against the average PGC number.

## Results

**Figure 1:**
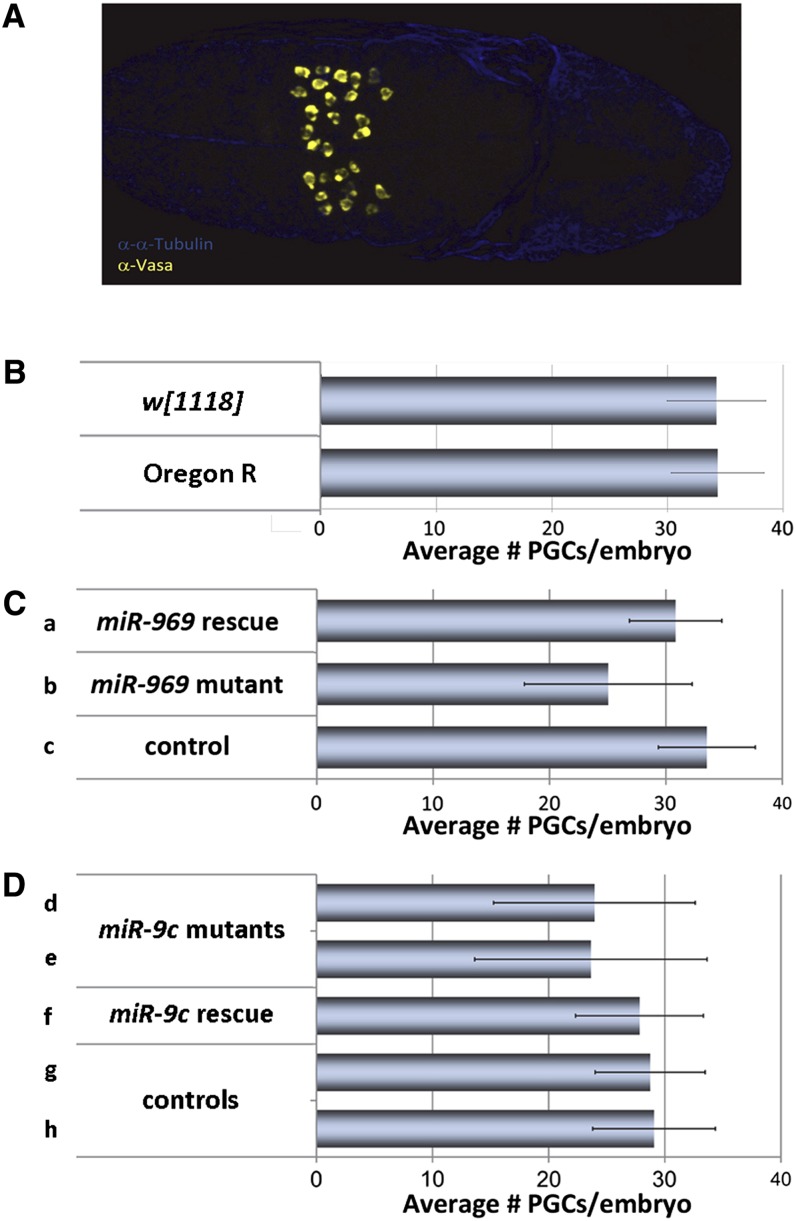
Maternal loss of *miR-969* and *miR-9c* leads to reduced primordial germs cell (PGC) numbers. (A) A wild-type embryo at developmental stage S10. PGCs are labeled with α-Vasa (yellow). At S10, PGCs are spread out dorsally toward the embryo surface. (B) Average PGCs numbers in two different laboratory control strains (± SD). (C) Average PGC numbers in embryos derived from *miR-969* mutant mothers are reduced in comparison with control and rescued embryos. Error bars correspond to the SD (± SD). Genotypes are (a) *Df(1)BSC352/miR-969^KI^;;T-969@Fb/+*, (b) *Df(1)BSC352/miR-969^KI^*, and (c) *Df(1)BSC352/+*. (D) Average PGC numbers in embryos derived from *miR-9c* mutant mothers are reduced in comparison with control and rescued embryos. Genotypes are (d) *miR-9c^KO^/miR-9c^KO^;T-9c@Fb/+*, (e) *miR-9c^KO^/miR-9c^KO^;+/nosGal4*, (f) *miR-9c^KO^/miR-9c^KO^;T-9c@Fb/nosGal4*, (g) *miR-9c^KO^/+;T-9c@Fb/+*, and (h) *+/miR-9c^KO^;+/nosGal4*

We used PGC numbers during embryonic stage S10 (Figure 1A) as a readout for the activity of maternal pathways that affect embryonic PGC development. Embryos of two different laboratory strains used as controls, Oregon R and *w^1118^*, had on average 34 PGCs per embryo at embryonic stage S10 (Figure 1B). We surveyed a collection of miRNA mutants for maternal effects on PGC numbers at S10. We observed that females lacking *miR-969* or *miR-9c* (Figure S1) produced S10 embryos with reduced PGC numbers (henceforth, we refer to embryos by the genotype of their mothers). The *miR-969* embryos had on average 25% fewer PGCs than heterozygous control embryos (Figure 1C; *P* < 0.001). Maternal expression of a *miR-969* transgene rescued this phenotype (Figure 1C; *P* < 0.001). Similarly, *miR-9c* embryos had ∼20% fewer PGCs than control embryos (Figure 1D; *P* < 0.001). Maternal expression of a *miR-9c* transgene restored PGC number to control levels (Figure 1D; *P* < 0.01). Therefore, maternal activity of these two miRNAs affects PGC development in the embryo.

**Figure 2:**
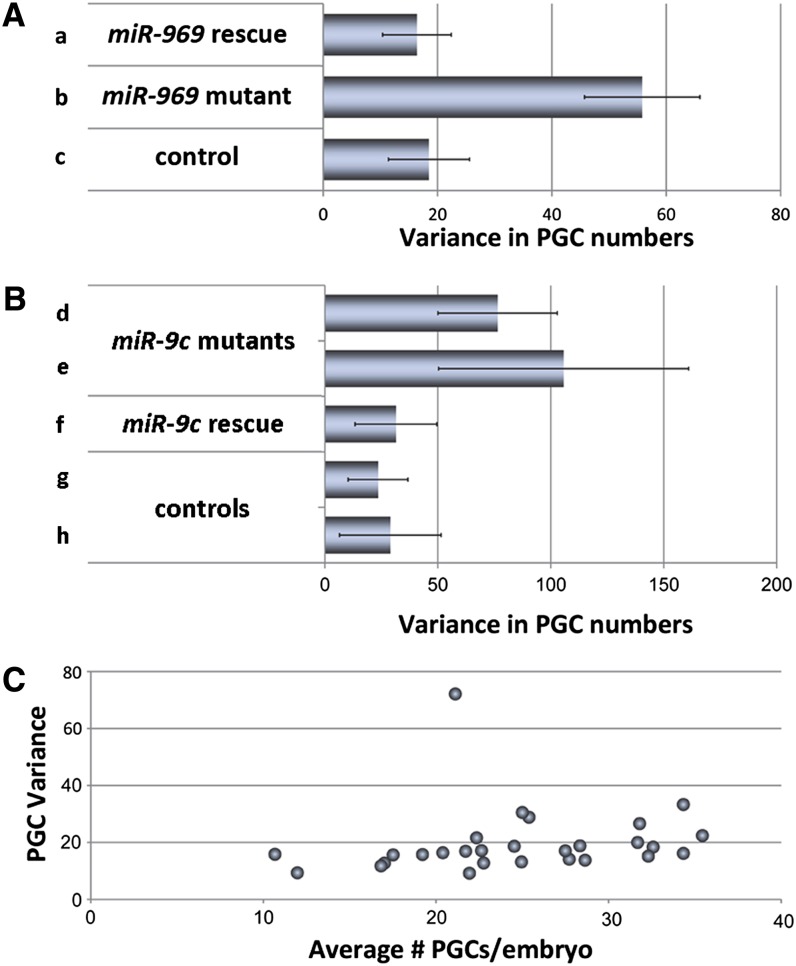
Maternal loss of *miR-969* and *miR-9c* leads to elevated primordial germs cell (PGC) number variance. (A) Average PGC number variance is elevated in embryos derived from *miR-969* mutant mothers in comparison with control and rescued embryos. Genotypes are (a) *Df(1)BSC352/miR-969^KI^;;T-969@Fb/+*, (b) *Df(1)BSC352/miR-969^KI^*, and (c) *Df(1)BSC352/+*. (B) Average PGC number variance is elevated in embryos derived from *miR-9c* mutant mothers in comparison with control and rescued embryos: (d) *miR-9c^KO^/miR-9c^KO^;T-9c@Fb/+*, (e) *miR-9c^KO^/miR-9c^KO^;+/nosGal4*, (f) *miR-9c^KO^/miR-9c^KO^;T-9c@Fb/nosGal4*, (g) *miR-9c^KO^/+;T-9c@Fb/+*, and (h) *+/miR-9c^KO^;+/nosGal4*. (C) Increased PGC number variance (*y*-axis) does not generally increase with reduced PGC numbers (*x*-axis). Data points were originally published in Kugler *et al.* 2010.

In addition, we noted that the range of PGC numbers was broader in embryos from the miRNA mutant mothers than from the controls. Some had normal numbers of PGCs, whereas others had very few. Quantification of this phenotype showed a clear increase in variance in PGC number (Figure 2, A and B; *P* < 0.001 for *miR-969*; *P* < 0.05 for *miR-9c*). Increased variance also was rescued by restoring maternal expression of the corresponding miRNA in the mutant background (Figure 2, A and B; *P* < 0.001 for *miR-969*; *P* < 0.05 for *miR-9c*). The magnitude of the variance observed in the rescued mutant embryos was comparable with that of the heterozygous mutant controls (Figure 2, A and B) and with that of the Oregon R and *w^1118^* control embryos.

We considered the possibility that increased PGC variance might be a phenomenon generally associated with reduction in PGC numbers. Genetic manipulation of the maternal activity of the ubiquitin E3 ligase specificity receptors Gustavus and Fsn has been shown to affect the stability of the germ plasm component Vasa, and can lead to moderate reduction in offspring PGC numbers (Kugler *et al.* 2010). We reanalyzed the original data from this study to ask whether reduced PGC number would correlate with increased PGC variance. Figure 2C plots the PGC number variance of all genotypes scored in this study (Kugler *et al.* 2010) against the average PGC number. Variance did not inversely correlate with PGC number.

## Discussion

miRNAs typically act on hundreds of target transcripts, and some miRNAs have been shown to stabilize regulatory networks, *e.g.*, *Drosophila miR-7* (Li *et al.* 2009). As such, miRNAs have been proposed to be ideal candidates to stabilize phenotypic traits by conferring robustness to developmental processes (Herranz and Cohen 2010; Ebert and Sharp 2012) or by canalizing development (Hornstein and Shomron 2006).

A priori, a microRNA reducing biological noise would be expected to decrease the variance of a quantitative trait without affecting the mean value. However, this assumes that the biological process allows for unconstrained variance equally in both directions. In the case of PGC production in *Drosophila*, many maternal effect mutations have been identified that cause reduced PGC numbers (Mahowald 2001; Lasko 2011), although we are not aware of any mutation that leads to increased PGC numbers. Increased PGC numbers have been observed on maternal overexpression of the germ plasm components *oskar*, *germ cell-less*, and *piwi* (Smith *et al.* 1992; Jongens *et al.* 1994; Megosh *et al.* 2006), but even mutations affecting translational repressors of *oskar* lead to reduced germ cell numbers (Ottone *et al.* 2012). This is likely due to additional functions such proteins have in establishing oocyte polarity and in *oskar* mRNA transport (Kugler and Lasko 2009). Taken together, this suggests that there may be constraints that limit variance in the direction of higher PGC number.

In the case of the two miRNAs studied here, we observed increased variance and a reduction in average PGC number. A correlation between a reduction in average PGC number and an increase in PGC number variance has been observed in four other maternal mutants (Yohn *et al.* 2003). Three of the mutants cause defects in the nuclear division pattern during syncytial embryogenesis and, hence, reduce the number of nuclei that associate with the germ plasm. The fourth impairs germ plasm assembly at an early step. We examined another dataset focused on two regulators of the germ plasm component Vasa, Gustavus and Fsn (Kugler *et al.* 2010), and did not observe a correlation between reduced germ cell number and increased variance. Although increased variance can accompany reduced PGC number, it appears likely that these are separable phenotypic traits.

Germ cell number depends on the coordination of many biological processes in the embryo, including nuclear division and migration and germ plasm production. The number of PGCs produced will depend on the amount of germ plasm and the number of nuclei that reach the periphery of the syncytial embryo at the correct time. Constraints on the number of nuclei that can enter the germ plasm or on the number that can migrate to the pole at the appropriate time could bias the potential for variance toward reduction of PGC number. Final PGC numbers also reflect the amount of proliferation and of loss due to apoptosis during migration. It also seems possible that variance in these processes might not be symmetric. It is possible *miR-969* and *miR-9c* act through multiple targets on these processes and that, through increased variance, lead to a reduction of PGC production, proliferation, or survival.

## Supplementary Material

Supporting Information
