# Supplementary material for: Maternal Loss of miRNAs Leads to Increased Variance in Primordial Germ Cell Numbers in Drosophila melanogaster
Source: G3 (Bethesda). 2013 Sep 1;3(9):1573–6. doi: 10.1534/g3.113.007591 (PMC3755917; doi:10.1534/g3.113.007591)
Supplement: Supporting Information [file supp_3_9_1573__index.html]

Maternal Loss of miRNAs Leads to Increased Variance in Primordial Germ Cell Numbers in Drosophila melanogaster — Supporting Information 

# Maternal Loss of miRNAs Leads to Increased Variance in Primordial Germ Cell Numbers in *Drosophila melanogaster*

## Supporting Information for Kugler *et al.*, 2013

**Files in this Data Supplement:**

- Supporting Information - Figure S1 and Table S1 (PDF, 2 MB)
- Figure S1 - Generation of *miR-9c* and *miR-969* knockout deletions through homologous recombination (PDF, 1 MB)
- Table S1 - Raw data: germ cell numbers in miRNA mutant and rescued embryos (.xls, 35 KB)
