# Supplementary material for: Maternal Loss of miRNAs Leads to Increased Variance in Primordial Germ Cell Numbers in Drosophila melanogaster
Source: G3 (Bethesda). 2013 Sep 1;3(9):1573–6. doi: 10.1534/g3.113.007591 (PMC3755917; doi:10.1534/g3.113.007591)
Supplement: Supporting Information [file supp_g3.113.007591_007591SI.pdf]

G3 · Genes | Genomes | Genetics

Maternal loss of miRNAs leads to increased variance in primordial germ cell numbers in *Drosophila melanogaster*

Jan-Michael Kugler[*,‡], Ya-Wen Chen[*], Ruifen Weng[*,§]
and Stephen M. Cohen[*,§]

*) Institute of Molecular and Cell Biology, Singapore 138673, Singapore
§) Department of Biological Sciences, National University of Singapore, Singapore 119613, Singapore
‡) E-mail: jmkugler@imcb.a-star.edu.sg

DOI: 10.1534/g3.113.007591

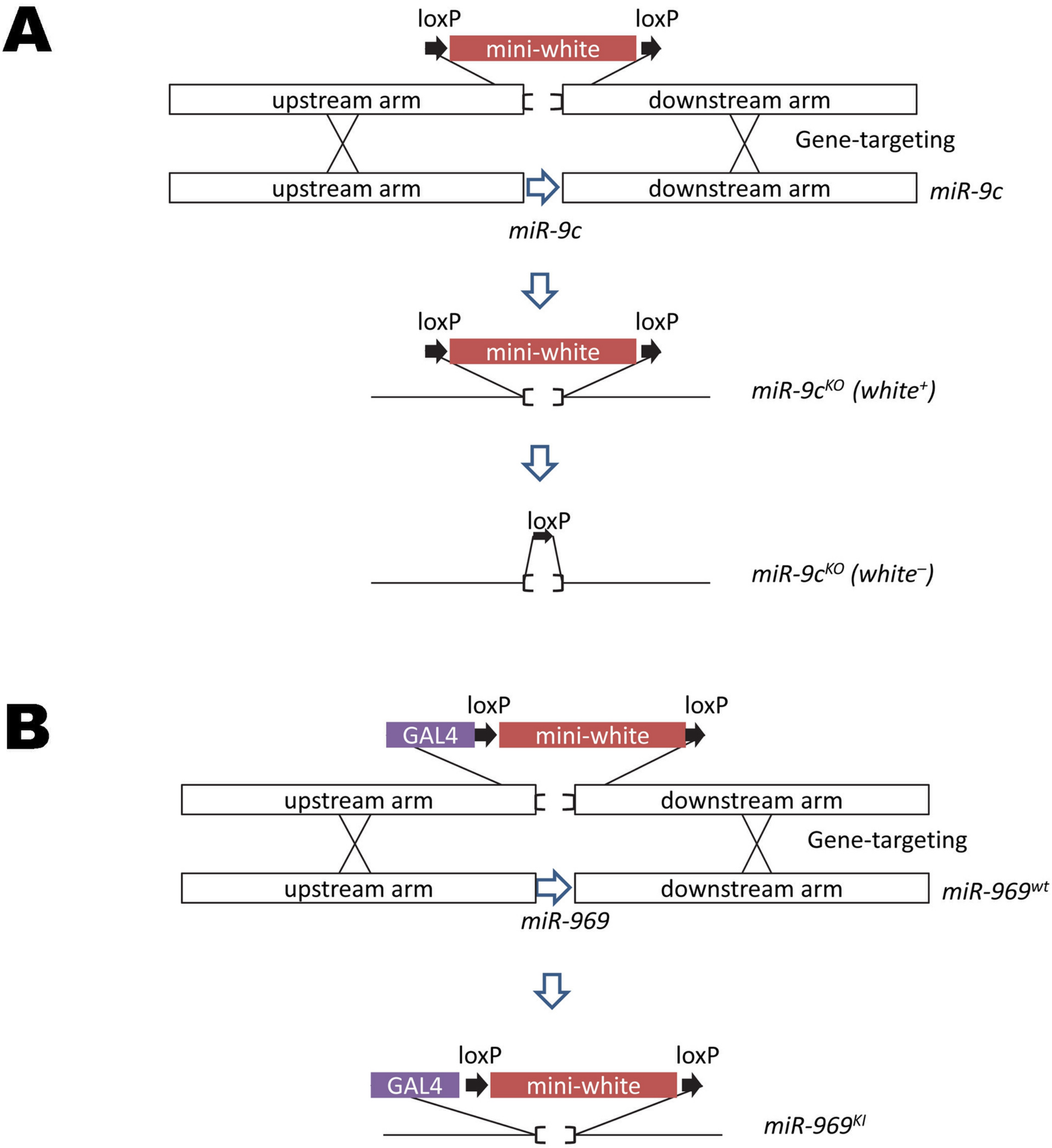

Suppl. Fig. 1: Generation of *miR-9c* and *miR-969* knockout deletions through homologous recombination

A. The endogenous *miR-9c* stemloop sequence was first replaced by a mini-white transgene through ends-out targeting. Afterwards, the mini-white transgene was removed by Cre-loxP-mediated recombination.

B. The endogenous *miR-969* stemloop was replaced by a Gal4 transgene, followed by mini-white. This places the Gal4 transgene under the control of the miR-969 promoter.

Table S1 Raw data: germ cell numbers in miRNA mutant and rescued embryos.

Table S1 is available for download at http://www.g3journal.org/lookup/suppl/doi:10.1534/g3.113.007591/-/DC1.
