# Supplementary material for: Maternal Loss of miRNAs Leads to Increased Variance in Primordial Germ Cell Numbers in Drosophila melanogaster
Source: G3 (Bethesda). 2013 Sep 1;3(9):1573–6. doi: 10.1534/g3.113.007591 (PMC3755917; doi:10.1534/g3.113.007591)
Supplement: Supporting Information [file supp_g3.113.007591_FigureS1.pdf]

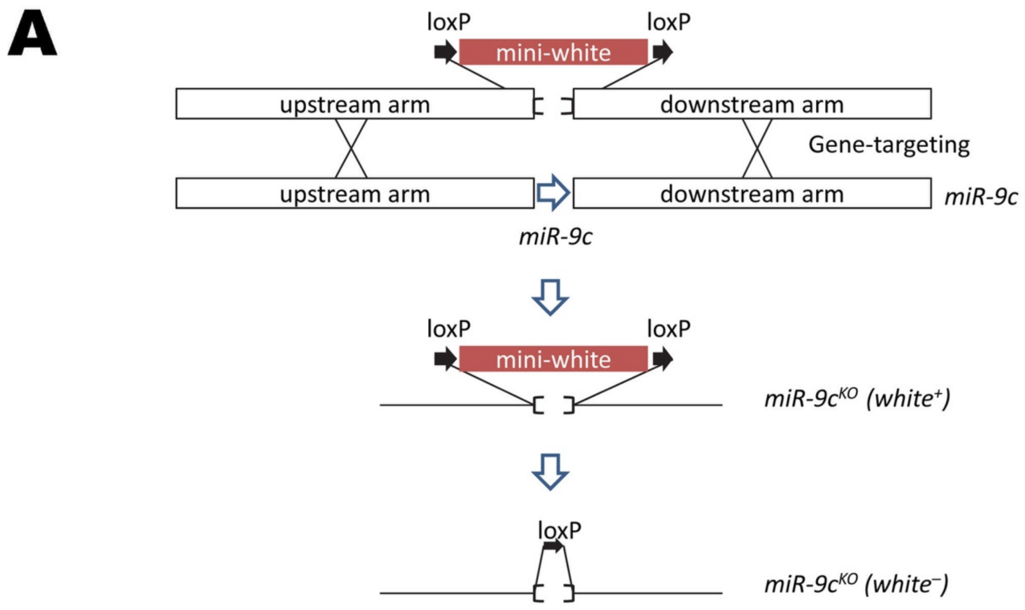

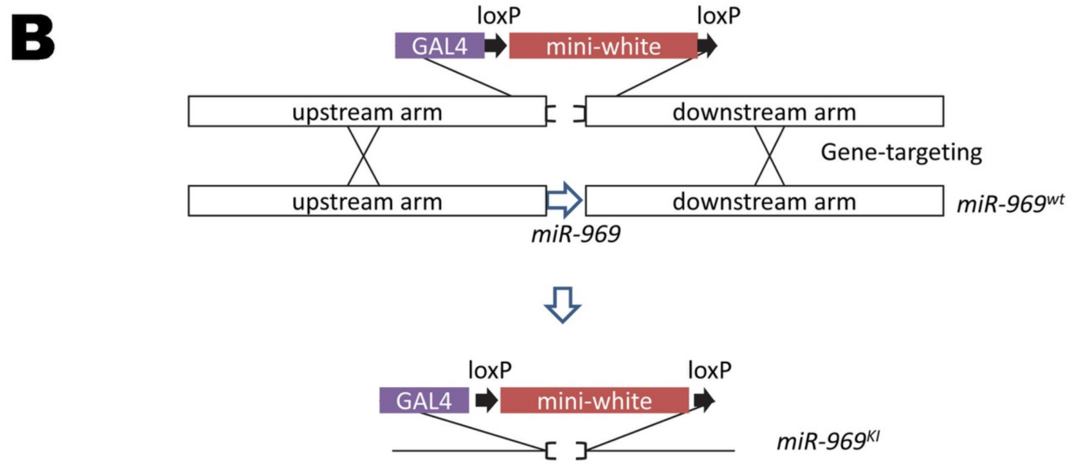

Figure S1 Generation of *miR-9c* and *miR-969* knockout deletions through homologous recombination

A. The endogenous *miR-9c* stemloop sequence was first replaced by a *mini-white* transgene through ends-out targeting. Afterwards, the *mini-white* transgene was removed by Cre-loxP-mediated recombination.
B. The endogenous *miR-969* stemloop was replaced by a Gal4 transgene, followed by *mini-white*. This places the Gal4 transgene under the control of the *miR-969* promoter.
